# Current Management of Locally Recurrent Rectal Cancer

**DOI:** 10.3390/cancers16233906

**Published:** 2024-11-21

**Authors:** Claudio Coco, Gianluca Rizzo, Luca Emanuele Amodio, Donato Paolo Pafundi, Federica Marzi, Vincenzo Tondolo

**Affiliations:** 1 UOC Chirurgia Generale 2, Fondazione Policlinico Universitario A. Gemelli IRCCS, 00168 Rome, Italy; claudio.coco@unicatt.it (C.C.); donatopaolo.pafundi@policlinicogemelli.it (D.P.P.); 2 UOC Chirurgia Digestiva e del Colon-Retto, Ospedale Isola Tiberina Gemelli Isola, 00186 Rome, Italy; lucaemanueleamodio@virgilio.it (L.E.A.); federicamarzi9@gmail.com (F.M.); vincenzo.tondolo@fbf-isola.it (V.T.)

**Keywords:** rectal cancer, local recurrence, adjuvant therapy, pelvic exenteration

## Abstract

Locally recurrent rectal cancer (LRRC), which occurs in 6–12% of patients previously treated with surgery, with or without pre-operative chemoradiation therapy, represents a complex and heterogeneous disease profoundly affecting the patient’s quality of life (QoL) and long-term survival. Its management usually requires a multidisciplinary approach, to evaluate the several aspects of a LRRC, such as resectability or the best approach to reduce symptoms. Surgical treatment is more complex and usually needs high-volume centers to obtain a higher rate of radical (R0) resections and to reduce the rate of postoperative complications. Multiple factors related to the patient, to the primary tumor, and to the surgery for the primary tumor contribute to the development of local recurrence. Accurate pre-treatment staging of the recurrence is essential, and several classification systems are currently used for this purpose. Achieving an R0 resection through radical surgery remains the most critical factor for a favorable oncologic outcome, although both chemotherapy and radiotherapy play a significant role in facilitating this goal. If a R0 resection of a LRRC is not feasible, palliative treatment is mandatory to reduce the LRRC-related symptoms, especially pain, minimizing the effect of the recurrence on the QoL of the patients. The aim of this manuscript is to provide a comprehensive narrative review of the literature regarding the management of LRRC.

## Simple Summary

This article comprises an extensive review of several aspects of locally recurrent rectal cancer (LRRC), which has a relatively low incidence, but its management represents a real challenge. The manuscript analyzed the main risk factors for the occurrence of an LRRC and evaluated the best diagnostic tools and the better staging systems. Moreover, this article analyzed and evaluated the multimodal management of an LRRC, with particular attention paid to surgical management and long-term oncological outcome after treatment.

## 1. Definition of Locally Recurrent Rectal Cancer

The term locally recurrent rectal cancer (LRRC) refers to the presence of a tumor in the lesser pelvis (also called true pelvis) after prior local or radical treatment for rectal cancer, including recurrences originating from lymph nodes [1]. Based on the type of management of the primary tumor, two distinct entities can be identified: true local recurrence of rectal cancer and local regrowth of rectal cancer. The term LRRC should be reserved for cases where a tumor reappears in the lesser pelvis following previous excisional surgical management of the primary rectal cancer. In contrast, local regrowth is more accurately used when a tumor reappears in the lesser pelvis without prior surgical excision after the establishing of a complete clinical response after CRT.

Furthermore, LRRC can be classified based on the type of surgical procedure performed. It may occur after radical resection (such as anterior resection or abdominoperineal resection) for rectal cancer, with partial mesorectal excision (PME, for intraperitoneal rectal cancer) or total mesorectal excision (TME, for extraperitoneal rectal cancer), or after local excision (LE). In the case of LE, two different subsequent surgical options should be considered. One option is completion TME (cTME), defined as a radical TME performed to prevent the occurrence of LRRC when high-risk pathological features are identified in the surgical specimen after LE. The other option is salvage TME (sTME), a radical TME performed, when possible, to treat LRRC that has occurred following a previous LE.

Finally, based on the concomitant use of adjuvant or neoadjuvant radiotherapy or chemoradiation therapy administered for the primary tumor, LRRC can be distinguished as occurring in a previously irradiated pelvic field or in a radiation-naïve pelvis.

## 2. Incidence and Risk Factors of LRRC

The management of primary rectal cancer requires a multidisciplinary approach, including surgery, chemotherapy, and radiotherapy. The introduction of mesorectal excision (PME or TME, depending on the site of the rectal tumor) and the optimization of preoperative neoadjuvant chemoradiation therapy (CRT) have significantly reduced the incidence of LRRC, which currently ranges between 6% and 12% [2,3,4,5].

Regardless of the initial management of the primary rectal tumor, the treatment of LRRC represents a significant challenge, and a multidisciplinary approach is essential due to the complexity of most recurrent disease cases. The first step is, of course, to prevent the onset of local recurrence by identifying risk factors and attempting to address modifiable ones. Many risk factors have been identified as predictors of increased risk for developing local recurrence. These can be categorized as patient-dependent, treatment-dependent, and tumor-dependent factors.

In terms of patient-dependent risk factors, patient constitution is one such factor. Patients with a narrow, “male” pelvis and obesity may present a more challenging surgical environment, potentially compromising adherence to the quality criteria necessary for radical oncological surgery for primary rectal tumors [6]. Patients with a compromised immune system, immunodeficiency disorders, or advanced age are also at a higher risk of developing LRRC [6].

Regarding treatment-specific risk factors, the presence of an institutional multidisciplinary board (MDT) of experts in rectal cancer care, along with a high volume of rectal cancer surgeries performed annually, serve as protective factors that reduce the risk of LRRC [1,7]. An annual number of 10–12 surgically treated rectal cancer cases per surgeon is considered adequate to certify experience and significantly reduce the rate of local recurrence compared to surgeons treating fewer patients [8]. Over the past 40 years, the introduction of total mesorectal excision (TME) and advancements in chemoradiation therapy regimens have profoundly transformed rectal cancer management, reducing the rate of local recurrence and improving survival and quality of life. Since Bill Heald’s introduction of TME in 1982, involving meticulous sharp dissection between the mesorectal and endopelvic fascia along an avascular, areolar “holy plane,” it has been recognized as a critical surgical procedure to significantly reduce local relapse, which, in the pre-TME era, ranged from 30% to 38% [9]. In Heald’s initial series analyzing the role of TME in rectal cancer, a significant reduction in local recurrence to 3.7% at 5 years was reported [9]. Subsequent series have shown that LRRC rates after TME are lower than 10% [10,11]. Current guidelines for rectal cancer treatment consider complete mesorectal excision mandatory in cases of extraperitoneal rectal cancer (mid and low rectum). For intraperitoneal rectal cancers (upper rectum), partial mesorectal excision (PME), maintaining a mesorectal margin of at least 5 cm, has been established as safe, yielding oncological outcomes comparable to TME while avoiding the risk of low colo-rectal anastomosis [12]. Thus, the quality of mesorectal excision and the status of the circumferential resection margin (CRM) are closely linked to the risk of local recurrence. The quality of TME can be classified as complete (intact mesorectum with only minor irregularities and no narrowing toward the distal margin), nearly complete (irregularities on the mesorectal surface and slight coning without exposed muscle), or incomplete (defects that extend to the muscle layer and an irregular mesorectal surface). Incomplete mesorectal resection is a significant risk factor for LRRC [13,14,15]. Nonradical resections (R1–R2) and positive circumferential resection margins (CRM+), defined as the closest radial margin between the deepest tumor penetration or an involved lymph node and the edge of the resected soft tissue around the rectum during mesorectal dissection, are strong predictors of LRRC. The most accepted definition of a negative CRM is a margin >1 mm from the tumor. CRM involvement is a strong predictor of local recurrence in both pre-operatively irradiated and non-irradiated patients. The pivotal study by Nagtegaal and Quirke, which analyzed 17,500 rectal cancer patients, demonstrated that CRM+ is a powerful predictor of both local and distant recurrence [16]. Another risk factor for LRRC is abdominoperineal excision (APE), which has been associated with a higher local recurrence rate (5–47%) compared to sphincter-saving anterior resection, likely due to a higher rate of positive CRM [17,18]. Recently, a more extensive APE technique, extra-levator abdominoperineal excision (ELAPE), which involves resection of the levator muscles en bloc with the mesorectum, has been introduced. ELAPE has been shown to result in a lower risk of involved CRM and fewer intra-operative bowel perforations [19,20].

An inadequate distal resection margin is also a risk factor for local recurrence. The risk of local recurrence decreases significantly as the distance between the tumor edge and the surgical margin increases, with benefits seen up to a distance of 5 mm of clear margin [21]. A positive microscopic margin is a strong risk factor for LRRC, with recurrence rates in such cases ranging from 31% to 55% [21]. Preoperative neoadjuvant radiation or chemoradiation therapy significantly reduces the rate of local recurrence in locally advanced extraperitoneal rectal cancer by improving resectability through tumor downstaging and downsizing [22,23,24]. In this context, the tumor response to neoadjuvant treatment is a critical prognostic factor for local control [25]. Over the past 30 years, there has been an increasing rate of pathological complete response (pCR) in rectal cancer patients following preoperative CRT. Achieving pCR, which currently occurs in approximately 20% of cases, is an important positive prognostic factor for local recurrence, disease-free survival (DFS), and overall survival (OS) [26,27].

The pathological TNM stage is the most well-known prognostic factor for the occurrence of LRRC, with a higher rate of recurrence in stage III compared to lower stages [28,29]. Other pathological factors influencing the prognosis of rectal cancer include the presence of lymph vascular or perineural invasion [30] and the presence of a bulky tumor [31]. In the multicenter study by Song et al., involving 1232 stage II-III rectal cancer patients, the presence of lymph vascular invasion—and especially perineural invasion—was significantly associated with a higher rate of LRRC [30,32,33]. In previously irradiated rectal cancer patients, the Tumor Regression Grade (TRG) pathological scoring system has been shown to be significantly correlated with the rate of locoregional lymph node metastasis and, consequently, with local control and disease-free survival. In the series by Vecchio FM et al., the 5-year local recurrence rate for TRG 1–2 was 2%, significantly lower than the 17% rate observed in TRG 3–5 [32]. In early rectal cancer treated by local excision alone, according to the most recent guidelines, T1 rectal tumors with high-risk pathological features (poor differentiation, lymph vascular infiltration, positive margins), greater than 3 cm in size, or involving more than 30% of the rectal circumference, are at risk for positive lymph nodes in the mesorectal fat and, consequently, for developing local relapse [12]. In such cases, a completion TME (cTME) is necessary to significantly reduce this high risk of local recurrence.

## 3. Symptoms of LRRC

Symptoms are often the first sign of LRRC, and at the time of diagnosis, between 16% and 66% of patients are symptomatic [34]. The most frequent symptoms of LRRC include weight loss, abdominal, pelvic, or back pain, tenesmus, bleeding, and changes in bowel function, up to and including obstruction. These symptoms are often refractory to medical therapy and can lead to a significant reduction in quality of life [34,35]. The presence of symptoms, particularly pain, at the time of LRRC diagnosis is considered a prognostic factor. Hahnloser et al. categorized patients based on the severity of their symptoms into three groups: S0 (asymptomatic), S1 (symptomatic, no pain), and S2 (symptomatic with pain). According to Hahnloser’s review, recurrence was asymptomatic (S0) in 23% of patients, symptomatic without pain (S1) in 23%, and symptomatic with pain (S2) in 54% of cases [36]. Symptomatic pain (S2) was significantly associated with unresectable LRRC and worse long-term survival. Without treatment, the mean survival time for LRRC is approximately 8 months, often accompanied by severe symptoms, particularly pain [36].

In addition to symptom analysis, a thorough medical history and physical examination can detect recurrence in 21% of cases [6].

## 4. Diagnosis of LRRC

High-quality imaging is essential for the successful management of LRRC. However, the anatomic pattern of tumor recurrence is highly variable, sometimes multifocal; moreover, in previously irradiated and surgically treated field, it is often difficult to distinguish between scar and neoplastic tissue, which represents a crucial aspect to establish the surgical resectability of the tumor [37].

In addition to the quality of instrumental diagnosis, another fundamental aspect in the treatment of LRRC is the early detection of recurrence. The best chance of cure for LRRC is closely related to early diagnosis at an early stage. In this context, it is essential to establish the pattern, duration, and frequency of follow-up after surgery for patients treated for primary rectal cancer who are at risk of recurrence (high-risk stage II and stage III rectal cancer).

Based on ESMO guidelines, during follow-up, clinical examination, completion of colonoscopy and pelvic imaging using MRI and/or CT and for distant metastases CT of the chest, abdomen, and pelvis are recommended [38]. A minimum provisional recommendation for average-risk patients is as follows:

- Clinical assessment: every 6 months for 2 years;
- Colonoscopy within the first year if not performed at the time of diagnostic work-up (e.g., if obstruction was present);
- Colonoscopy with resection of colonic polyps every 5 years up to the age of 75 years;
- A minimum of two CTs of the chest, abdomen and pelvis in the first 3 years and regular serum CEA tests (at least every 6 months in the first 3 years) [38].

A high-intensity surveillance in rectal cancer patients at risk for recurrence offers significant benefits, including earlier detection of LRRC, more opportunities for curative reoperation, and significantly improved overall and disease-free survival. Higher-intensity surveillance groups report a greater frequency of recurrences (18.9% vs. 6.3%) and more curative resections (10.7% vs. 5.7%) [39]. However, these advantages must be balanced against potential drawbacks, such as more invasive testing, financial costs, and psychological stress [34]. To mitigate these drawbacks, it is crucial to classify specific recurrence patterns and stratify patients based on risk factors. Tumor-related risk factors may include disease stage, invasion of other structures, tumor grading and fixation, mucinous tumor components, and previous adjuvant treatments [6]. Individual risk factors may include patient comorbidities, activity level, age, patient preferences, and compliance [34].

To determine the duration of follow-up, it is important to note that both local and distant recurrences are detected in 62% of cases within the first 2 years, 80% within 3 years, and 92% within 4 years. After 5 years, recurrence rates drop to less than 1.5% per year and to 0.5% per year after 10 years [40]. Periodic evaluation of symptoms and clinical visits for patients treated for primary rectal cancer should be performed in conjunction with CEA-level blood measurements. The frequency of follow-up may be every 3–6 months during the first 2 years and every 6 months up to 5 years [34]. Clinical visits are essential, as symptoms often signal recurrence, with 16% to 66% of patients being symptomatic at the time of diagnosis [41,42]. A digital rectal examination should be able to detect the presence of intraluminal or perirectal recurrence near the site of the primary surgery, and a biopsy of the suspected lesion should be performed, possibly with an anoscopy. The clinical significance of CEA level measurement during follow-up depends on whether the pre-operative CEA level was elevated, with sensitivity and specificity for recurrence detection ranging from 43% to 98% and from 70% to 90%, respectively [6].

According to ASCRS guidelines, in high-risk patients for developing cancer recurrence, a CT scan of the chest, abdomen, and pelvis should be performed annually for 5 years [34]. CT scanning is considered the gold standard for diagnosing LRRC, with a sensitivity of around 76% [43], though it has a significant rate of false positives [44]. Magnetic resonance imaging (MRI) and fluorodeoxyglucose positron emission tomography (FDG-PET) may be helpful in cases of diagnostic uncertainty, but their routine use is not recommended due to their limitations [1,38]. T2-weighted MRI has high sensitivity (80–91%), specificity (86–100%), and accuracy (95%) in detecting LRRC, and diffusion-weighted imaging (DWI) can further improve accuracy [45,46,47]. However, its positive predictive value varies, as differentiating between postoperative changes, fibrosis, and tumor tissue can be challenging [46,48]. FDG-PET may be useful in distinguishing scar tissue from a viable tumor, with reported accuracy for LRRC ranging from 74% to 96% [49], although it is limited in detecting small lesions, mucinous tumors, and positive lymph nodes, particularly after radiochemotherapy [50].

Follow-up proctoscopy is recommended every 6–12 months for 3–5 years [34], with more frequent follow-up for high-risk patients, such as male patients, those with distal lesions, close margins, or poor response to neoadjuvant chemoradiation [51,52,53,54,55].

A biopsy is mandatory to confirm the diagnosis of LRRC, but it can be technically challenging, especially for extraluminal recurrences. In these cases, an endorectal ultrasound-guided biopsy should be performed to increase the likelihood of a diagnostic sample [56].

In rectal cancer patients treated with an organ-sparing approach, whether through non-operative management or local excision after a major or complete response to preoperative chemoradiation therapy, strict follow-up is necessary to rapidly identify (and treat) local regrowth or recurrence. Studies adopting this approach recommend proctoscopy every 3 months during the first 2 years and pelvic MRI every 6 months during the first 2 years [57,58].

During the last years, circulating tumor DNA (ctDNA) has also been increasingly used as a noninvasive biomarker in the follow-up of rectal cancer patients, to detect the occurrence of recurrent disease [59]. In the recent meta-analysis conducted by Nassar et al. on studies analyzing the role of ctDNA on rectal cancer (on 1022 patients), patients with positive pre-operative ctDNA status had more than four times the risk of developing a LRRC (compared with no ct-DNA patients); moreover, patients with positive postoperative ctDNA status had more than eight times the risk of developing a LRRC (compared with ct-DNA patients), underlining the potential role of ctDNA in stratify a population of rectal cancer patients at risk for a local relapse [60].

## 5. Staging System

Planning an adequate treatment for LRRC is based on an accurate staging of the recurrence. Although several classification systems exist, LRRCs are generally staged according to the anatomical location of the relapse, as surgical resectability depends largely on the site of recurrence and its relationship with surrounding structures. The MSKCC anatomical classification of LRRC categorizes recurrences as follows [61]:

Axial recurrences: Confined to the pelvic organs without invading the bone or sidewall, including anastomotic recurrences after low anterior resection, recurrences after local excision procedures, and perineal recurrences after abdominoperineal resection (APR).

- Anterior recurrences: Involving genitourinary organs.
- Posterior or sacral recurrences: Invading the sacrum.
- Sidewall or lateral recurrences: Invading iliac vessels, pelvic autonomic nerves, pelvic ureters, or extending through the greater sciatic foramen.

According to this anatomical classification, resectability was found to be highest in axial tumors compared to lateral tumors. The rate of R0 resection varied by location, with axial recurrences at 85.2%, anterior at 33.3%, posterior at 25%, and lateral at 4.3% (*p* < 0.001) [62].

The Royal Marsden classification system (established also by Beyond TME Group) further subdivides the anterior location into “anterior above the peritoneal reflection,” “anterior below the peritoneal reflection” (involving the genitourinary system and pubic symphysis), and the “anterior urogenital triangle” (involving the perineal body/scar, vaginal orifice, distal urethra, and crus of the penis). Additionally, this system introduces the “infraelevator” location, which includes the levator ani muscles, external sphincter complex, and ischio-anal fossa [63,64]. According to this classification, the central location represents the most frequent site of LRRC (18%) and patients with LRRC in the anterior location above the peritoneal reflection had a poorer overall survival compared with other sites of recurrence [63,64].

Another classification system is the Wanebo et al. classification, which is based on a modified TNM staging system as follows [65]:

- TR1 and TR2: Intraluminal local recurrence at the primary resection site.
- TR3: Anastomotic recurrence with full-thickness penetration beyond the bowel wall into the perirectal fat tissue.
- TR4: Invasion into adjacent organs, including the vagina, uterus, prostate, bladder, seminal vesicles, or presacral tissues, with tethering but not fixation.
- TR5: Invasion into the bony ligamentous pelvis, including the sacrum, lower pelvic sidewalls, or sacrotuberous-ischial ligaments.

An alternative classification system, known as the Suzuki or Mayo Clinic classification system, categorizes LRRCs by both the site of onset and the degree of fixation to surrounding organs and symptoms [66]:

- FO: No fixation.
- F1: Fixation at one site.
- F2: Fixation at two sites.
- F3: Fixation at three or more sites among the following four: anterior adjacent organs, right or left lateral pelvic sidewalls, and posterior sacrum or coccyx.
- S0: Asymptomatic.
- S1: Symptomatic without pain.
- S2: Symptomatic with pain.

These criteria relate to the horizontal spread of recurrent tumors, with patients whose tumors are confined to the perineum (caudal spread) or small bowel (cephalad spread) classified as Stage FO [66].

In the historical series by Suzuki et al., the 3-year and 5-year overall survival (OS) was high in FO patients (61.5% and 50%, respectively), but progressively lower in patients with F > 1. OS was also influenced by the presence of symptoms: in S0 cases, the 3-year and 5-year OS were 68.4% and 37.2%, respectively, while in S2 cases, these rates were significantly lower (31.6% and 16.3%). Additionally, the degree of pelvic fixation in LRRC was strongly related to the risk of postoperative morbidity, ranging from 14% in FO patients to 44% in F3 patients [66].

In 2020, the National Cancer Institute of Milan (NCIM) developed a new staging system as follows [67]:

- S1a: LRRC located axially within the rectal stump or anastomotic wall (intraluminal relapse).
- S1b: Rectal or pararectal localization without invasion of regional organs.
- S1c: Involvement of adjacent anterior genitourinary organs.
- S2a: Sacral involvement below S2.
- S2b: Sacral involvement at the S1-2 level.
- S3: Lateral pelvic wall involvement.

In a study by Sorrentino et al., comparing the predictive power of several staging systems in 152 consecutive patients with LRRC, the NCIM classification system proved superior to others in predicting R0 resections and, more importantly, in predicting non-R0 resections, which were significantly higher in S2b and S3 LRRCs [68].

## 6. Management of LRRC

The prognosis of LRRC depends on the possibility of curative treatment. If left untreated, the prognosis is poor, with a median survival of 6 to 7 months, often complicated by symptoms such as pelvic pain refractory to standard analgesia, malodorous discharge, and incontinence [69,70,71]. Palliative management with radiotherapy or chemoradiation therapy alone rarely achieves complete tumor regression, especially in previously irradiated patients, but it can help control pelvic pain and prolong survival to 6 to 8 months. However, this palliative treatment is often accompanied by toxic side effects [72,73].

Therefore, surgical treatment remains the primary therapeutic approach to either cure or to providing adequate palliation of LRRC-related symptoms. In recent years, advancements in surgical techniques, reconstructive options, complementary therapies, and the management of operative complications have contributed to making surgery for LRRC safer and more feasible, improving postoperative outcomes, quality of life, and long-term oncologic results [74]. However, surgical resection for LRRC is a highly challenging procedure, often requiring multi-organ resections in a field that has been previously operated on, irradiated, and re-irradiated.

To maximize the chances of achieving radical treatment for LRRC, the involvement of a multidisciplinary team is crucial. This team should include colorectal, urological, gynecological, neurosurgical, orthopedic, and plastic surgeons, as well as radiologists, oncologists, pathologists, stoma care nurses, social workers, and case managers, all with extensive experience in pelvic exenteration [75]. The role of the multidisciplinary team in the treatment of LRRC was evaluated by Zhao and colleagues, who reported an R0 resection rate of 87.8% in patients deemed radically resectable by a multidisciplinary board [62].

### The Concept of Resectability

Radical resection of LRRC, defined as the ability to achieve a microscopically clear margin (R0) with acceptable postoperative morbidity and mortality, is the most important prognostic factor for the long-term oncologic outcome of patients [33,69,76,77,78,79,80]. Paradoxically, while the introduction of total mesorectal excision (TME) has significantly reduced the incidence of LRRC, it has also made excision more complex due to the extension of the surgical resection plane outside the “holy plane,” reducing the possibility of achieving an R0 resection [81].

The R0 resectability of LRRC should be determined based on an analysis of the following:

- Tumor-related features, through preoperative imaging that precisely describes tumor location and the degree of local invasion, as well as the patient’s history of the primary rectal tumor, including TNM stage, previous APR, and high carcinoembryonic antigen levels.
- Patient-related features, such as comorbidities, male sex, advanced age, and the presence of pain [63].

The location and degree of local invasion of the tumor are considered the primary factors in determining the resectability of LRRC. The following features of LRRC are considered contraindications for primary surgical management:

- Poor patient performance status.
- Unresectable extrapelvic disease.
- Proximal sacral invasion extending to the sacral promontory (above S2–S3).
- Tumor extension through the greater sciatic notch.
- Encasement of the external iliac vessels.
- Presence of lower limb edema due to lymphatic or venous obstruction.
- Bilateral hydronephrosis caused by ureteric obstruction.
- Extensive circumferential involvement of the pelvis (especially sidewall involvement).

The presence of metastatic disease is common in patients with LRRC (up to 50%), but this does not necessarily preclude surgical intervention if the extra-pelvic disease is deemed resectable (either synchronously or after primary surgery) and if the patient is fit for an extensive surgical procedure [82]. The PelvEx collaborative group described also the feasibility of simultaneous pelvic exenteration and liver resection in a group of 128 oligometastatic (2 cm) LRRC patients [83]. An R0 resection was achieved in 73.5% of cases with a rate of 30-day mortality and morbidity of 1.6% and 32%, respectively [83]. The 5-year overall survival in R0 patients was 54.6%, significantly better than R1–2 patients (20%) [83].

Although sacral involvement at the level of S1 and S2 is considered a relative contraindication, it is often associated with involvement of the common iliac vessels or ureter, which represent absolute contraindications to surgery [84].

Circumferential involvement of the pelvic wall is also a contraindication due to the poor oncologic prognosis when three or more pelvic sites are involved, as indicated by the Suzuki classification system [66].

Regarding hydronephrosis as a contraindication, it is important to distinguish the level of ureteric obstruction. When obstruction occurs high in the pelvis or along the lateral sidewall, achieving an R0 resection is more challenging; however, when it is distally located near the bladder, a curative resection may still be possible [85,86].

According to available data, only 40–50% of all patients with LRRC can be expected to undergo surgery with curative intent, and of those, 30–45% will achieve an R0 resection [1]. Thus, only 20–30% of all patients with LRRC will have a potentially curative operation [1]. However, the resectability rate for LRRC can be improved with the administration of neoadjuvant preoperative chemoradiation therapy or by using intraoperative radiotherapy (IORT) [87,88].

Thanks to the improvement in concomitant chemoradiation therapy, the rate of radical resection increased during the last years. In the first large series of PelvEx Collaborative group (2004–2014) on 1184 LRRCs treated by surgery (and neoadjuvant treatment in 78.1% of cases) the rate of R0 resection was 55.4%, with a bone resection rate of 20.3% and flap reconstruction rate of 17.4% [89]. In the following series (2017–2021) on 800 LRRCs treated by surgery (and neoadjuvant treatment in 81.5%) the rate of R0 resection, bone resection and flap reconstruction increase, respectively, to 71.7%, 41.9%, and 32.1%, significantly better than the previous rates [90]. The improvement in resectability rates over the years has also led to improved survival rates, as documented by the recent study from the Beyond TME Collaborative group on 2996 patients with LRRC (locally recurrent rectal cancer) who underwent pelvic exenteration (R0 rate 83%). The 5-year survival of patients who underwent surgery after 2005 was significantly higher than those who had surgery before 2005 (61.7% vs. 37%), even in the R0 resection subgroup (62.1% vs. 40.6%) [91].

## 7. Neoadjuvant Treatment

In primary rectal cancer, preoperative neoadjuvant chemoradiation therapy (CRT) has played an important role in improving oncologic outcomes and increasing the resectability of unresectable, locally advanced rectal cancer. This evidence has also been demonstrated in the management of LRRC.

Re-irradiation (re-RT) plays a role in increasing the rate of radical resection or in the definitive treatment of inoperable patients. In the multicenter study by Holman et al. [92], involving 251 LRRC patients, the rate of R0 resection in irradiated patients ranged from 43% to 50%, significantly higher than the R0 rate of 26% recorded in non-irradiated patients. Additionally, preoperative radiotherapy improves local control following R0 resections. In a study by Ogawa et al., the 5-year recurrence-free survival after R0 resection was significantly higher in irradiated patients (24.4%) compared to non-irradiated patients (0%) [93].

The trouble concerning re-RT is related both to the received dose of the organs at risk (OARs) and to the best time between the two irradiations. Moreover, administering a suboptimal dose to control the side effects can result in failure to control or downstage and downsize the disease [88,94]. However, the great progress in radiation treatment allows highly conformal treatments to be delivered to the target site, avoiding organs at risk. Moreover, radiation therapy is increasingly moving toward the use of new technologies such as carbon ion RT (CIRT), proton therapy (PBR), and MR-Linac-guided adaptive RT. A recent Italian systematic review (on 7 studies, 230 patients) evaluated the effects of these modern techniques in the management of LRRC [95]. The study reported promising rate of OS (90% and 73.0%, respectively, at 1-year and 2-years) and local control (89.0% and 71.6%, respectively, at 1-year and 2-years). The overall rate of the G3 acute toxicity ranged from 0% to 22.7% and the more frequent toxicity was acute G3 gastro-intestinal toxicity (0–13.6%). The overall rate of G3 late toxicity ranged from 0% to 37.7% and the most frequent G3 late toxicity was gastrointestinal (0–19.3%) [95].

In the setting of neoadjuvant chemoradiation therapy (CRT) for LRRC, it is necessary to distinguish patients who previously received radiation therapy (70%) from patients who are RT-naïve (30%) [2]. Also, in the setting of LRRC, the rationale of pre-operative CRT is based on the potential capacity of CRT in inducing a downstaging and a downsizing with consequently higher possibility to perform an R0 resection and to obtain, in about 10% of cases, a complete tumor response [2]. In both RT-naïve and pre-irradiated patients, radiotherapy is associated with capecitabine (825 mg/m^2^, bidaily) or fluorouracil-based chemotherapy [1,38].

In RT-naïve patients, several studies demonstrated the positive effects of full-course neoadjuvant CRT (25 × 2Gy or 28 × 1.8 Gy) in obtaining an R0 resections and in improving the oncological outcomes of LRRC [1,38]. In the study of Bosman et al., the rate of R0 resection after neoadjuvant CRT was 63% and in previously treated patients the local recurrence-free survival and overall survival were better than patients undergone to up-front surgery (respectively 70% vs. 35% and 50% vs. 32%) [89]. In line with the study of Bosman et al., Dijkstra and colleagues reported an analogous rate of R0 resection (68%) after neoadjuvant CRT with acceptable rates of 5-year overall survival (32%) and disease-free survival (26%) [96].

The efficacy of pre-operative CRT was demonstrated also in previously irradiated LRRC patients especially in obtaining and increasing the rate of radical resection In the study of Bosman et al. [97] and in the study of Owens and colleagues [98], the preoperative CRT was associated to a higher rate of R0 if compared with patients underwent to up-front surgery (respectively 56% vs. 42% and 43% vs. 26%) even if no differences were found about oncologic outcome. Moreover, the series by Sun et al. reported the role of neoadjuvant CRT also in increasing the resectability of unresectable LRRC reporting a rate of R0 resection of 25% [99]. The improving techniques of chemoradiation therapy especially in the sparing of organ at risk significantly reduce the toxicity related to neoadjuvant therapy, with a decreasing of interruptions from 30% to 4% and with a decreasing of grade 3 toxicity under 10% [2,96]. In previously irradiated LRRC patients, the use of hyperfractionation seems to be able to increase the tumor dose without increasing late toxicity [87]. Actually, the use of CRT in RT-naïve LRRC patients and the use of conventional fractionation up to 30 Gy in previously irradiated LRRC patients are recommended by the ESMO and Beyond-TME guidelines [1,38] and the ESTRO-ACROP recommendations [100].

Similar to primary rectal cancer, in LRRC previously treated with CRT, the occurrence of a pathological complete response (pCR) is a favorable prognostic factor. In a large Dutch series by Nordkamp et al., a pCR occurred in 51 of 345 patients (14.8%) and was associated with 3-year overall survival (OS), disease-free survival (DFS), and local re-recurrence-free survival rates of 77%, 56%, and 82%, respectively. These were significantly better than the rates for patients without pCR (51.1%, 26.1%, and 44%, respectively) [101]. These results, recorded also in LRRC, suggest the possibility of adopting an organ-sparing approach in highly selected cases of complete responders after CRT. However, further studies are needed to confirm the feasibility and safety of this approach.

The promising results described in the AZUR-1 trial, a prospective phase-2 trial of 16 patients with mismatch repair-deficient (dMMR) rectal cancer treated by immunotherapy which obtained a 100% of clinical complete response, led to a rise in interest in immunotherapy [102]. However, no evidence about the use of immunotherapy exists but it is likely that immunotherapy could be beneficial also for dMMR LRRC patients.

Also, in the field of LRRC, it is mandatory to analyze the biology of cancer to test its responsivity to chemotherapic drugs and to personalize the target of the therapy. All LRRC patients must be tested for DNA mismatch repair deficiency (MMR-d) by immunohistochemistry staining for mismatch repair proteins MLH1, MSH2, MSH6, and PMS2 or microsatellite instability (MSI) by polymerase chain reaction or NGS. MMR-d or MSI-high (MSI-H) tumors are associated with a decreased response to 5-fluorouracil-based chemotherapy, an enhanced response to immunotherapy, and in general have an improved prognosis compared with MMR-proficient tumors (MMR-p) [103,104]. Moreover, MMR-d/MSI, KRAS, NRAS, BRAF, and Her2 should be tested in all patients with metastatic LRRC, regardless of age at diagnosis, to guide therapy selection [105].

## 8. IORT

In the context of LRRC, where the tumor may be fixed to the bone and muscular structures of the pelvis, intraoperative radiotherapy (IORT) appears to assist surgeons in reducing or delaying the risk of local re-recurrence after surgery with close or positive margins (R1–2 resections).

In the large meta-analysis by Mirnezami et al., involving 1211 LRRC patients, the addition of an IORT boost improved oncologic outcomes in terms of local control (*p* = 0.03), disease-free survival (*p* = 0.009), and overall survival (*p* < 0.001), with only an increase in wound complications [106]. A recent study by Ansell J et al. compared the outcomes of patients treated for LRRC with an R1 resection converted to R0 resection after re-resection to those who remained R1 after resection and were treated with IORT. No differences in overall survival were found between the two groups (*p* = 0.62), suggesting that the use of IORT in patients with persistent R1 had the same effect as re-resection, likely due to low tumor volume being adequately treated by high doses of radiation [107]. In a Mayo Clinic series on LRRC patients who underwent R2 resection, the role of IORT was demonstrated by increasing 3-year overall survival (44% vs. 15% without IORT) and reducing the rate of 3-year local relapse (40% vs. 93% without IORT) [108]. In 2020, the ESTRO-ACROP guidelines recommended IORT as a boost after preoperative chemoradiation therapy for patients with potentially resectable tumors and for those undergoing debulking surgeries, with a dose of 12.5 to 15 Gy (in R0) and 15 to 20 Gy (in R1 and R2) to the area at highest risk of subsequent local relapse [100].

## 9. Surgical Treatment

Surgery is the most important treatment for achieving a radical cure of LRRC. In LRRC surgery, it is necessary to distinguish between resections confined to the relapse (involving the pelvis and neorectum) that do not require the removal of other pelvic organs, and extended radical resections, which involve the removal of at least one adjacent pelvic organ (such as the bladder, prostate, uterus, vagina, ovaries, ureter, iliac vessels, small bowel, or sacrum).

The best position for the patient during surgery for LRRC is the Lloyd–Davies position, although the prone jackknife position may be necessary in certain cases. An initial thorough exploration of the abdominal cavity is essential to exclude occult disease, such as peritoneal carcinomatosis, which is a contraindication for curative resection [106].

If the relapses involve other organs, an en bloc resection with cancer-free margins should be performed. If there are concerns about margin status, a frozen-section examination of the suspected area should be performed to assess the need for additional surgical enlargement [109]. The extent of resection is guided by the site of the relapse.

For axial LRRC, if the relapse is located at the level of the anastomosis or within the mesorectum and does not extend into the anterior genitourinary structures, an anterior resection (if feasible, depending on the distance between the recurrence and the anorectal ring) or an abdominoperineal excision should be performed. These procedures are more complex compared to the same procedures for primary rectal cancer due to the need for unusual dissection planes to achieve an R0 resection.

For anterior LRRC, if the tumor involves urogenital structures, extended radical resection is necessary. In women, if the uterus and/or vagina are involved, a posterior exenteration (removal of the anus/neorectum, vagina, uterus, salpinges, and ovaries) is recommended. If the recurrence involves the dome of the bladder, a concomitant en bloc partial cystectomy should be performed. Similarly, if the recurrence involves the prostate alone, an en bloc bladder-preserving prostatectomy should be performed. However, involvement of the bladder trigone by LRRC necessitates a total pelvic exenteration. Ureteral reconstruction may be performed using either ileal or colonic conduits.

For posterior LRRC involving structures posterior to the anus/neorectum (such as the presacral fascia and sacrum), extended radical resection, including resection of sacral or perisacral structures, is required. If bony invasion is present, sacral resection is necessary to achieve an R0 resection. Reconstruction of the pelvic floor in these cases can be challenging, and the use of omentum, absorbable mesh, or pedicle flaps may be necessary to prevent small bowel herniation. Involvement of the sacrum above the S2-3 junction presents a surgical challenge and is often considered a contraindication for surgery. However, the multicentric study by Lau et al. analyzed oncologic outcomes after 345 high (at or above the S2–S3 junction) and low (below the S2–S3 junction) sacrectomies for primary and recurrent rectal cancer. The overall rate of R0 resection in LRRC was 63.4%, and no significant differences were observed between high and low sacrectomies in terms of 5-year overall survival (53% vs. 44.1%) and 5-year cancer-specific survival (60% vs. 56.1%), indicating that, if an R0 resection is achievable, infiltration of the high sacrum should not be considered a contraindication for surgery [110]. However, the rate of postoperative morbidity after en bloc sacrectomy for LRRC is significant. A systematic review by Sasikumar et al. found that the length of hospital stay and the rate of major complications increased with more proximal sacral transections, from 45% after low sacrectomy to 61% after high sacrectomy [111]. If the invasion is limited to the sacral fascia or periosteum, these structures can be resected without requiring sacrectomy. This approach may allow for a radical resection in cases extending to S1 and S2 without the need for high or total sacrectomy. In a series by Shaikh et al., the technique of high subcortical sacrectomy for disease extending to the upper sacrum at S1 and S2 was evaluated in five patients to avoid high or total sacrectomy or a nonoperative approach. All patients had an R0 resection, with only one patient experiencing a major complication, and no postoperative deaths were recorded within 90 days [112].

Lateral recurrences, involving the lateral pelvic wall, are associated with a high rate of iliac vessel and ureter involvement and have the lowest likelihood of achieving a radical resection. When resection is feasible, it carries a high risk of bleeding and intraoperative complications [113,114]. In lateral LRRCs, structures that may be involved, from superior to deep layers, include the autonomic nerves, ureters, internal iliac system, lumbosacral trunk, sacral nerve roots, and sciatic nerve. Partial or complete resection of the sciatic nerve involved in LRRC has been described by Brown and colleagues, reporting a 65% R0 resection rate and a 76% 5-year local recurrence-free survival rate. Additionally, 90% of patients retained independent mobility [114]. Major vascular resections are often necessary to achieve extended radical resection. Abdelsattar and colleagues reported an R0 resection rate of 58.3% in LRRC involving major vessels (including internal, external, and common iliac vessels), requiring vascular reconstruction (using synthetic interposition grafts, femoral–femoral bypass, or primary anastomosis) with an acceptable rate of major morbidity and no graft-specific complications. The 4-year oncologic outcomes were promising, with overall survival (OS) and disease-free survival (DFS) rates of 55% and 45%, respectively [115]. The feasibility of combined vascular excision and reconstruction has also been described by Brown et al., who reported a 38% R0 resection rate, a vascular-related morbidity rate of 52%, and a median DFS of more than 2 years in LRRC with major vessel involvement [116]. Extensive vascular resections with graft reconstruction pose a risk of graft infection due to bowel or genitourinary tract handling during surgery. These procedures are also associated with prolonged operative times and an increased risk of lower limb ischemia during the perioperative period. In cases of lateral LRRC, Austin et al. reported on a large series of 100 patients who underwent en bloc resection of pelvic sidewall structures, including the internal iliac vessels, piriformis and obturator internus muscles, ischium, and sacrotuberous and sacrospinous ligaments. The rate of R0 resection was 62%, with promising long-term oncologic results (5-year OS rate of 35%). However, there was a significant rate of postoperative morbidity (82%), particularly in patients with iliac vessel resection, and a reoperation rate of nearly 25% [116,117,118]. Extended pelvic resections often result in a hollow pelvic cavity, which can lead to major complications such as pelvic abscesses, fistulas, bowel obstructions, and poor perineal wound healing—referred to as “empty pelvis syndrome” [119]. The administration of adjuvant therapy (chemotherapy or chemoradiation therapy) in the pelvic bed can impair perineal wound healing [119].

A widely accepted approach to address this issue is the use of tissue with a vascular pedicle to fill the empty space and close the perineal wound. Perineal defects can be closed primarily with omentoplasty, biological or absorbable mesh, or by using a pedicle flap (such as a transpelvic rectus abdominis myocutaneous flap, gracilis myocutaneous flap, or gluteal myocutaneous rotational or advancement flaps) [65,120]. The use of a flap to close perineal defects has been shown to be superior to primary closure, with or without pedicle omentoplasty, in terms of time to closure and rates of perineal wound dehiscence, although it significantly prolongs operative time and requires the involvement of a plastic surgeon [121,122,123,124].

## 10. Outcomes

After radical surgery, disease relapse can occur as metastatic disease and/or local re-recurrence. In a large systematic review of 55 cohort studies (1990–2010) involving 3767 patients treated for LRRC, the observed crude rate of distant metastases during follow-up varied between 9% and 68%, with a median rate of 41%. Among patients who achieved an R0 resection, the crude local control rate ranged between 29% and 100%. The 5-year survival rate ranged from 11% to 51% [4].

In a Swedish national population-based study of 426 patients with LRRC treated with surgery alone, surgery with curative intent was performed in 149 patients (35%), with radical surgery achieved in 64 patients (R0 resection rate of 53%). Non-centrally located tumors were more likely to have positive resection margins (R1/R2). The 5-year survival for patients resected with curative intent was 43% after R0 resection and 14% after R1 resection [125].

A recently published systematic review and meta-analysis, involving 4744 patients, of which 42.5% had LRRC [126], reported an R0 resection rate ranging from 14.9% to 77.8% [126]. This rate of R0 resection varies with different treatment approaches for LRRC. A large review analyzing data on oncological outcomes for different treatment modalities in LRRC patients (15 studies, 974 patients: 346 undergoing neoadjuvant radiotherapy, 279 neoadjuvant CRT, 136 adjuvant CRT, 189 surgery alone, and 24 surgery with IORT) found a higher R0 resection rate in the neoadjuvant CRT group (64.07%) compared to neoadjuvant radiotherapy (52.46%) and adjuvant CRT (47.0%) [35].

As previously mentioned, achieving an R0 resection is the most important factor predictive of good oncologic outcomes. In a French multicenter study (29 French surgical center), radical resection was achieved in 60% of cases, and surgical radicality was associated with a better 5-year overall survival rate (60%) compared to R1 (29%) and R2 resections (11%) [127]. Similarly, in a multicenter Japanese study of 498 patients with LRRC, a favorable 5-year overall survival rate (52%) was recorded for patients who underwent R0 resection, which was feasible in 42.8% of cases [128].

In a study comparing outcomes after surgery alone and surgery following chemoradiation therapy, the neoadjuvant CRT group had the highest mean 3-year survival (51.41%) compared to surgery alone (46.8%), neoadjuvant radiotherapy (42.06%), and surgery with IORT (25.0%) [36,129,130,131]. Neoadjuvant CRT also demonstrated better local control compared to neoadjuvant radiotherapy (49.5% vs. 22.0%, respectively) and surgery plus IORT (57.90% vs. 37.60%, respectively). No studies reported local control rates for patients treated with surgery alone [129,130,131].

In the Netherlands series by Hagemans et al., involving 193 LRRC patients who underwent surgery, 90% received preoperative chemoradiation therapy, and R0 resection was achieved in 60% of cases [132].

## 11. Postoperative Morbidity and Mortality

The postoperative complication rate depends on the type of surgery performed and increases with more complex and extensive procedures. The rate of significant postoperative morbidity therefore varies considerably, ranging from 15% to 68% in different studies [69,77,80,109]. Pelvic abscess formation and wound infections are common, but other types of complications, including wound breakdown, anastomotic leakage, ileus, pneumonia, pulmonary embolism/deep vein thrombosis, and sepsis, are not rare. Postoperative mortality is typically around 3%. In the large systematic review by Tanis and colleagues, the mean postoperative mortality rate was 2.2% [4].

In the Swedish national population-based study, 62 patients (41.6%) experienced a complication within 30 days after tumor resection. Patients who received surgical treatment without tumor resection had a lower complication rate but a significantly higher 30-day mortality rate than those who underwent tumor resection (10% versus 1.3%, respectively; *p*: 0.002) [133]. In the Netherlands series by Hagemans et al., an R0 resection was achieved in 60% of cases after preoperative chemoradiation therapy, with short-term postoperative mortality and morbidity rates of 3% and 34%, respectively. The most common complications were wound related, and re-intervention was necessary in 13% of cases [132].

In a recent systematic review on pelvic exenteration, the median length of hospital stay after surgery ranged from 9.3 to 29.1 days, with a median ICU stay ranging from 2.0 to 3.3 days [126]. The reported rate of short-term postoperative complications was higher than 50% (52%), with gastrointestinal complications and wound infections being the most frequent (15.9% and 19.9%, respectively) [126]. A significant percentage of patients (14.1–45.5%) required unplanned hospital readmission. The median rate of short-term postoperative mortality was 1.7% (range: 0–8.7%) [126].

The recent large series by PelvEx and Beyond TME collaborative groups reported a rate of 30-day postoperative mortality after pelvic exenteration respectively of 1.8% and 4.6% [90,91]. In the PelvEx study the overall 30-day postoperative morbidity rate was 58.8% with a rate of major morbidity of 23.1% [90].

Regarding quality of life (QoL), most QoL metrics returned to baseline (or were only slightly lower) at 12 months post-surgery. However, the physical component scores deteriorated more in younger patients compared to elderly patients, likely because younger patients had greater initial physical ability before pelvic exenteration, which declined after surgery [126].

## 12. Quality of Life

An important outcome measure in the treatment of LRRC is represented by QoL. Surgical treatment remains the best chance of cure for LRRC but often a multi-visceral resection or a pelvic exenteration is needed to obtain an R0 resection, with a potential risk of worsening the patient’s QoL [90]. Despite the extension of surgical resection in LRRC, previous studies have demonstrated a reasonable QoL in patients undergoing surgery for LRRC with a trend of slow increase from the third month post-operations [134]. The study of Huang Y et al. compared the QoL after pelvic exenteration in patients affected by primary locally advanced rectal cancer (111) and in patients affected by LRRC (160). Nevertheless, patients with LRRC had lower rates of R0 resection, longer length of stay, longer operative time, and shorter median overall survival than patients affected by primary rectal cancer; no significant differences were found between two groups about QoL outcomes at any time points postoperatively up to 12 months [135]. Moreover, the recent multicenter study of Harji DP and colleagues analyzed the short-term health-related quality of life in LRRC underwent to surgery or to palliative treatments. The median time of comparative evaluation was 4 months. Health-related QoL, according to the Functional Assessment of Cancer Therapy—Colorectal cancer (FACT-C) score, was better in patients undergoing to surgical treatments and, in particular, in patients undergoing to R0 resection (compared with R1–2 patients) [136].

Achieving a balance between oncological outcomes and patient quality of life should be the primary objective of multidisciplinary treatment for LRRC [90]. This balance, however, cannot be reached without an adequate patient counseling process, which assesses the methods and risks of extremely radical surgery and whose outcome is represented by the shared therapeutic decision with the patient [91]. About this aspect, several clinical studies have demonstrated the positive prognostic role of active patient involvement and prehabilitation before surgical treatment [137,138,139]. Such outcomes also appear achievable with the help of PROMs (patient-reported outcome measures), which, by collecting data on the patient’s postoperative QoL, assist surgeons in assessing the impact of radical surgery on the patient’s QoL [137,138,139]. In this setting, the PelvEx Collaborative, in collaboration with the European Organization for Research and Treatment of Cancer network, has developed and supported a project aimed at creating a specific PROM tool for quality of life, with the ultimate goal of achieving survival with an acceptable QoL [90]. A similar project has been developed by the Beyond TME Pelican Cancer Foundation, the IMPACT (Improving Management for Patients with Advanced Colorectal Tumors) program, which highlighted the importance of patient involvement in the decision-making process, incorporating functional and quality outcomes as key measures of oncological success [140].

## 13. Palliative Treatment

Palliative treatment for locally recurrent rectal cancer focuses on alleviating symptoms and improving the quality of life for patients who are not candidates for curative treatment. Recurrence of rectal cancer can lead to severe symptoms, including pain due to nerve or bone involvement, bowel obstruction, urinary or fecal incontinence, and bleeding. The goal of palliative care in this context is to manage these symptoms through various interventions tailored to the patient’s needs and overall condition. Options to improve the patient’s quality of life and relieve the symptoms are radiation, local minimally invasive treatment, and surgery.

Radiation therapy is one of the most common palliative options, particularly for pain relief and control of bleeding. This treatment can also help reduce tumor size, which may alleviate obstructions or other pressure-related symptoms [120]. Radiation treatment seems to reduce LRRC-related pain and bleeding in almost 75% of patients [141]. Several studies analyzed the role of palliative radiation therapy both on primary and locally recurrent rectal cancer. A large systematic review analyzing the main 1990s studies reported an overall response rate of 78% for pain, 81% for bleeding and discharge, and 71% for mass effect [142]. More recent studies, adopting most modern 3-dimensional radiation techniques reported response rates of 86.7% for bleeding, 82.1% for pain, and 62.5% for obstruction [143].

A clear relationship between radiation dose and symptom response has been demonstrated. In the study of Wong et al., 48% of patients with pain responded after a total dose of less than 20 Gy, 77% after a dose of 20–30 Gy, 79% after 30–45 Gy, and 89% after 45 Gy or more [71]. In LRRC, Wong et al. reported that the percentage of patients with controlled symptoms for almost six months increased with dose (12% with 21–30 Gy, 31% with 31–40 Gy, and 58% with 41–50 Gy) [71]. Also, in a palliative field, the question of re-irradiation represents a criticism due to the increased risk of re-treatment toxicity. In the study of Mohiuddin et al. on 103 patients 22% had grade 3 toxicity and 6% had grade 4 toxicity, suggesting a re-irradiation dose according to the interval between previous RT and re-RT as follows: 35 Gy for an interval of 3–12 months, 40–45 Gy for 12–24 months, 45–50 Gy for 24–36 months, and 50–55 Gy for more than 36 months [144].

In cases of obstructing unresectable disease, the use of self-expanding metallic stents can be effective in temporarily relieving blockage. While stents are highly successful in many cases, with success rates around 97%, some patients may experience complications such as stent migration or pain, particularly if the obstruction is located near the anal verge. So, a considerable number of patients (18%) will require surgical palliation due to stent failure [145]. Malignant obstruction by LRRC in the last 5 cm of the anal verge is considered a relative contraindication for the use of rectal stents because of foreign body sensation and pain even if acceptable results were reported also when stents are used in patients with distal rectal obstructions [146].

When the use of a stent is not indicated, surgical palliation is necessary. The creation of a diverting defunctioning stoma can be performed in patients with unresectable LRRC suffering from disabling symptoms [145,146]. In highly selected patients with symptoms refractory to minor surgical approach, the decision to undergo major palliative surgery could be considered on a case-by-case basis [147]. Intractable symptoms, especially pain or bleeding, could require palliative pelvic exenteration after a clear discussion with patients about all risks and benefits [148]. About this aspect the PelvEx collaborative study group performed a systematic review on 23 studies and 509 patients undergone to palliative pelvic exenteration [149]. The most common indications for extensive surgery were pain, symptomatic fistula, bleeding, malodor, obstruction and pelvic sepsis. The postoperative morbidity and mortality rate were, respectively, 53.6% and 6.3% for a median overall survival of 14 months [149]. However, major surgery was able to obtain symptom relief in a median of 79% of patients, leaving an option open for extensive surgery in a highly selected group of patients [149].

Overall, the management of locally recurrent rectal cancer in a palliative setting requires a multidisciplinary approach, involving surgeons, oncologists, radiation therapists, and palliative care specialists, to provide personalized treatment plans aimed at symptom control and improving the patient’s remaining quality of life.

## 14. The Criticism of Low- and Middle-Income Countries

In developing countries, there are significant challenges in adopting and implementing a multidisciplinary approach for the treatment of cancer and, in particular, of rectal cancer and LRRC. In many of these countries, the lack of standard diagnostic facilities, imaging techniques, innovative surgical techniques, and adequate implementation of pathological examination. This causes delays in diagnosis and increased mortality due to the locally advanced stage at which the disease often presents [150].

The Lancet Commission on Global Cancer Surgery emphasizes the need for universal access to safe and affordable surgical cancer care, building capacity in surgical oncology, implementing research, and investing in cost-effective surgical care strategies to improve cancer outcomes in LMICs [151,152]. However, one of the main barriers for colorectal cancer patients in low- and middle-income countries is the high cost of essential chemotherapy drugs, which reduces their already limited availability and significantly restricts their use. Access to palliative care is also severely compromised due to the scarce availability of opioid drugs in these countries. Furthermore, access to targeted therapies and immunotherapy drugs—treatments that have significantly changed the clinical trajectory of colorectal cancers in recent years—is almost nonexistent. Access to radiotherapy treatments is even more challenging, as they require highly specialized centers that are generally unaffordable for these countries [150].

Improving the infrastructure for diagnosis, surgery, medical and radiation oncology, and ensuring access to essential chemotherapy drugs and palliative cancer care is necessary to provide optimal treatment for colorectal cancer patients in developed countries and should be incorporated into national health policies with adequate funding. Multidisciplinary tumor boards (MDTs), essential for improving diagnostic and therapeutic accuracy in cancer treatment, should also be implemented in national policies for these countries, as they have been shown to improve cancer-related survival, enhance the quality of life of cancer patients, and serve as a valuable teaching platform for junior doctors and residents [153].

The use of MDTs is currently even more accessible thanks to artificial intelligence and the implementation of web networks, which allow for virtual collaboration between physicians and boards in developing countries with physicians and multidisciplinary boards in Western countries. This could help to optimize the diagnostic–therapeutic process for the patient.

The NCCN has published resource-stratified guidelines for CRC to help national healthcare systems provide optimal care for patients with limited resources by prioritizing cost-effective interventions that can improve the quality and effectiveness of healthcare systems in LMICs [154].

## 15. Conclusions

The treatment of local recurrences of rectal cancer remains one of the greatest challenges faced by surgeons. These patients require a holistic approach in specialized centers dedicated to this type of problem, where a close-knit multidisciplinary team can design the most tailored therapeutic path for each individual patient. These operations often carry a high rate of complications and can result in significant functional consequences. Nonetheless, R0 surgery remains the only curative options for these patients and its feasibility rate is increasingly higher, thanks to improvements in neoadjuvant treatments, the trend toward performing increasingly complex radical and reconstructive surgeries, and the adoption of a more tailored therapy approach. This aims to achieve a balance between the aggressiveness of the treatment and the patient’s quality of life.
